# ACT001 synergizes with temozolomide-based chemoradiotherapy to cure refractory glioblastoma by targeting TNF-CXCL10-CD8^+^ T-cell immunity

**DOI:** 10.3389/fphar.2026.1745656

**Published:** 2026-02-12

**Authors:** Ya Shu, Ze-hua Ding, Pan-pan Gao, Qi An, Li-qin Wu, Xin-ran Zhang, Hai-feng Jiang, Cheng Miao, Mei-ting Peng, Xiao-qian Chen, Jun Cai, Feng Liu

**Affiliations:** 1 Department of Pharmacy, The First Affiliated Hospital of Yangtze University, Jingzhou, China; 2 Department of Oncology, The First Affiliated Hospital of Yangtze University, Jingzhou, China; 3 Department of Pathophysiology, School of Basic Medicine, Tongji Medical College, Key Laboratory of Neurological Diseases, Ministry of Education, Hubei Provincial Key Laboratory of Neurological Diseases, Huazhong University of Science and Technology, Wuhan, China; 4 The First Maternity and Infant Health Hospital, Tongji University, Shanghai, China; 5 Department of Respiratory and Critical Care Medicine, Zhongnan Hospital of Wuhan University, Wuhan, China

**Keywords:** ACT001, CD8, chemoradiotherapy, C-X- motif chemokine 10, glioblastoma, TNF

## Abstract

Glioblastoma multiforme (GBM), a highly invasive brain tumor, is severely restricted in T-cell infiltration and anti-tumor activity due to its immunosuppressive microenvironment. However, commonly used preclinical GBM mouse models cannot fully recapitulate the refractoriness of human GBM or effectively distinguish therapeutic efficacy. In this study, we evaluated the efficacy and mechanisms of therapies based on the novel sesquiterpene lactone small-molecule compound, ACT001, using the refractory G422^TN^-GBM mouse model. ACT001 alone exerted evident anti-G422^TN^-GBM effects *in vivo* and *in vitro*, but it only slightly prolonged animal survival. ACT001 combined with concurrent radiotherapy and temozolomide (RT/TMZ) exerted synergistic effects by suppressing tumor progression and extending animal survival. Importantly, the RT/TMZ/ACT001 regimen could achieve cure (long-term survival, >100 d, 26.7%) and immune cure (passing the tumor-rechallenge assay, >100 d, 12.5%) in G422^TN^ mice. However, combining the anti-PD-1 antibody (αPD-1) with RT/TMZ/ACT001 did not further improve survival. Mechanistically, RT/TMZ/ACT001 substantially activated the tumor necrosis factor (TNF) pathway, inducing tumor cells and stromal cells in the microenvironment to express the chemokine C-X-C motif chemokine 10 (CXCL10), thereby promoting T-cell infiltration, especially CD8^+^ T cell, into the tumor site. Pharmacological inhibition of the TNF signaling pathway with R-7050 completely abolished the synergistic efficacy of RT/TMZ/ACT001. Taken together, our results demonstrate that ACT001 combined with RT/TMZ can overcome the immunosuppressive barrier of GBM to achieve immune cure in GBM via TNF-CXCL10-CD8^+^ signaling, strongly suggesting the priority of combining ACT001 with RT/TMZ rather than with αPD-1 in clinical trials.

## 1 Introduction

Glioblastoma multiforme (GBM) is the most common and aggressive primary malignant brain tumor, pathologically characterized by rapid growth and a poor prognosis (Tan et al., 2020; Wu et al., 2021; Xiong et al., 2024). Usually, GBM patients undergo surgical resection, followed by radiotherapy and chemotherapy as the standard treatment approach (Singh et al., 2025). Temozolomide (TMZ) remains a first-line treatment drug, but given that GBM patients are prone to developing drug resistance, the median survival period is only 15 months (Krex et al., 2007; Johnson and O’Neill, 2012). The latest research indicates that preoperative combination therapy using PD-1 (nivolumab), CTLA-4 (ipilimumab), and LAG-3 (relatlimab) inhibitors has been employed to release multiple inhibitory brakes on T cells, achieving a 17-month recurrence-free survival (Long et al., 2025). However, it has not been verified on a large scale in clinical practice. Therefore, there is an urgent need to develop new therapeutic drugs in combination with conventional therapies.

ACT001 is certified as an orphan drug by the Food and Drug Administration (FDA) in the United States (Zhang et al., 2012; An et al., 2015) and is also known as dimethylamino-micheliolide (DMAMCL), a natural sesquiterpene lactone isolated from *Michelia compressa* (Viennois et al., 2014; Liu Q. et al., 2020; Jaffar et al., 2021). In the treatment of GBM, ACT001 penetrates the blood–brain barrier (BBB), is usually synergized with cisplatin (Xi et al., 2019), and prolongs survival in preclinical models. Phase II/III trials for recurrent GBM are ongoing (Lickliter et al., 2021; Zhang et al., 2022; Li et al., 2023), including a biomarker-driven trial (STAT3-high signature) (Clinical Trial NCT06894225). Directly binding to plasminogen activator inhibitor-1 (PAI-1), ACT001 could suppress PI3K/AKT signaling and inhibit tumor proliferation, invasion, and metastasis (Xi et al., 2019). Another anticancer mechanism is inhibiting STAT3 phosphorylation, reducing PD-L1 transcription, and reversing immunosuppression (Van Loo and Bertrand, 2023). The mechanism by which ACT001 inhibits the progression of glioma is not well understood, which limits the application of this new therapeutic target.

The tumor necrosis factor (TNF) signaling pathway plays an important role in inflammation and the immune response to tumors. It can kill tumor cells itself and reshape the tumor immune microenvironment (van Loo and Bertrand, 2023). After tumor necrosis factor alpha (TNFα) binds to tumor necrosis factor receptor 1 (TNFR1) on the surface of glioma cells, it triggers extrinsic apoptosis via the TRADD–FADD–caspase8 cascade (Zhou et al., 2020). The expression level of TNFR1 in glioblastoma (GBM) is positively correlated with TNF sensitivity (Ding et al., 2015; Alshevskaya et al., 2024). RIPK1 is a key protein in Complex I (where TNFα binds to its receptor TNFR1); studies have shown that palmitoylation modification is the key switch that activates the kinase activity of RIPK1 (Alshevskaya et al., 2024). Activated RIPK1 can trigger the following cell death programs: the apoptosis pathway, the necroptosis pathway, and iron-mediated cell death sensitization (Lafont et al., 2018; Yuan et al., 2019), which can strongly inhibit the proliferation of tumor cells. In particular, in melanoma, combined stimulation with TNFα and interferon-gamma (IFN-γ) can increase the expression of CXC chemokine ligand 10 (CXCL10) by more than 10-fold (Flood et al., 2022).

In this study, the orthotopic G422^TN^-GBM model used has been established as a treatment-refractory model that recapitulates the poor response to standard temozolomide and radiotherapy observed in human recurrent GBM (Liu F. et al., 2020). Although previous therapeutic explorations in this highly refractory G422^TN^-GBM model have focused on agents such as the ROS inducer piperlongumine (PL) or the CD73 inhibitor AB680 combined with RT/TMZ (Liu et al., 2023; Gao et al., 2025), the potential of targeting the immunomodulatory TNF signaling pathway to achieve a durable immune-mediated cure remains unexplored. In this study, we used this model to investigate whether ACT001, a compound with reported anti-inflammatory properties, could provide a novel therapeutic approach by activating the TNF pathway. We evaluated its efficacy alone and in combination with RT/TMZ, with the specific aim of verifying whether such a strategy could not only extend survival but also remodel the immunosuppressive microenvironment to achieve long-term immune-mediated cure—an outcome that has not been reported with PL or AB680 in this setting. Our results demonstrate that ACT001 effectively inhibited glioma progression, reshaped the immune microenvironment via the TNFα–CXCL10–CD8^+^T-cell axis, and led to a marked extension of overall survival, including instances of immune cure, in G422^TN^-GBM-bearing mice.

## 2 Materials and Methods

### 2.1 Animals

Adult male Kunming mice (4 weeks old, 18–22 g) were purchased from Hubei BIONT Biological Technology Co., Ltd. and housed at the Animal Center of Tongji Medical College, Huazhong University of Science and Technology (HUST), under a 12-h/12-h light/dark cycle with free access to food and water. All animal handling and experimental procedures were conducted in accordance with the NIH guidelines, the ARRIVE guidelines, and the regulations of the Animal Care and Ethics Committee of Tongji Medical College, HUST.

### 2.2 Cell and orthotopic model

The murine glioblastoma (GBM) cell lines G422^TN^-GBM and GL261 were generously provided by Prof. Chen’s team at Tongji Medical College, HUST. The highly invasive G422^TN^-GBM cell line was established and characterized through repeated *in vivo* passages; whole-genome sequencing confirmed that its genotype matches that of human IDH wild-type, triple-negative GBM, which is associated with a highly refractory phenotype and a poor response to standard therapies such as temozolomide. GL261 cells were cultured in DMEM (11965092, Gibco, NY, United States) supplemented with 10% fetal bovine serum (FBS, 10270106, Gibco, NY, United States). To preserve its tumorigenicity and aggressive phenotype, the G422^TN^-GBM cell line was maintained exclusively through *in vivo* passaging in syngeneic Kunming mice as previously described (Liu et al., 2023; Gao et al., 2025). An orthotopic, highly refractory GBM model was established by stereotaxic implantation of freshly isolated G422^TN^-GBM cells into syngeneic Kunming mice, following the protocol described by Liu F. et al. (2020) and Liu et al. (2023). In brief, mice were anesthetized via an intraperitoneal injection of 350 mg/kg chloral hydrate (C1048, Sigma-Aldrich, St. Louis, MO, United States) and 10 mg/kg xylazine (X1126, Sigma-Aldrich, St. Louis, MO, United States). A suspension containing 2 × 10^4^ viable G422^TN^-GBM cells in 1 µL of phosphate-buffered saline (G4202-500ML, ServiceBio, Wuhan, China) was drawn into a 10-µL Hamilton syringe (Hamilton Company, United States). Using a stereotaxic frame (RWD Life Science, China), the cell suspension was microinjected into the right striatum of the mouse brain at the following coordinates relative to bregma: 0.5 mm anterior, 2.0 mm lateral, and 3.5 mm ventral from the skull surface. The injection was performed at a constant rate of 1 μL/min using a microsyringe pump (KD Scientific, United States). The needle was left in place for an additional 6 min after injection to minimize backflow and ensure precise delivery. Animals were monitored post-operatively, and any showing signs of intracranial hemorrhage or severe neurological deficits within 24 h were excluded from the study. Mice were randomly assigned to experimental groups using a computer-generated randomization sequence. Throughout the experiment, the investigator performing bioluminescence imaging and survival endpoint assessments was blinded to group allocation.

### 2.3 ACT001, RT/TMZ, R-7050, αPD1, AB680, and piperlongumine treatment

ACT001 was provided by Accendatech Co., Ltd. (ACT001-1, Accendatech, Tianjin, China) and stored at −20 °C protected from light until experimentation. For administration, it was diluted in saline at a final concentration of 20 mg/mL or 40 mg/mL and administered to mice (200 mg/kg or 400 mg/kg) daily via oral gavage (Tong et al., 2020). R-7050 (TNF receptor antagonist, HY-110203, MCE, Shanghai, China) was dissolved in a solvent mixture (10% DMSO, 40% PEG 300, 5% Tween-80, and 45% saline) and administered orally at a dose of 5 mg/kg. TMZ (M2129, AbMole, Shanghai, China) was dissolved in 0.5% CMC-Na and administered orally at a dose of 50 mg/kg. The treatment regimen comprised an initial 5-day administration period, a 2-day drug-free interval, and a final 5-day administration period (Liu et al., 2023). For radiotherapy (RT, whole-brain irradiation), a single dose of 10 Gy was delivered using a biological X-ray irradiator (RS2000pro, Rad Source Technologies, United States) with fixed parameters (225 kV, 12 mA, and 1 Gy/48.4 s). αPD1 (clone RMP1-14, BE0146, Bio X Cell, New Hampshire, United States) was diluted in sterile PBS to 1 mg/mL and administered via intraperitoneal injection with an initial dose of 400 μg/mouse, followed by subsequent doses of 200 μg/mouse every other day for a total of six doses (Liu et al., 2023). AB680 (CD73 inhibitor, HY-125286, MedChemExpress, New Jersey, United States) was dissolved in a solvent mixture (5% DMSO, 40% PEG 300, 5% Tween-80, and 50% saline; Sigma-Aldrich, United States) and administered orally at a dose of 1 mg/mL and via intraperitoneal injection, following the protocol described by Gao et al. (2025). Piperlongumine (ROS inducer, SML1556, Selleck Chemicals, China) was dissolved in a mixture (10% Tween-80 and 90% saline) at a final concentration of 0.5 mg/mL and was administered to mice (5 mg/kg) daily via intraperitoneal injection, according to the previous therapeutic schedule (Liu et al., 2023). All treatments were initiated on day 7 after tumor cell implantation, with a 3-h interval between drug administrations on the same day. Animals were monitored daily for clinical signs, including hunched posture, lethargy, and difficulty ambulating. Humane endpoints were applied, and euthanasia was performed if severe symptoms developed.

### 2.4 *In vivo* experimental design and statistical considerations

The sample size for animal studies was predetermined based on our previous experience with the G422^TN^-GBM model (Liu F. et al., 2020; Liu et al., 2023) and power analysis to ensure adequate statistical power. For survival and longitudinal bioluminescence imaging experiments, a group size of eight mice (n = 8–9) was used. For endpoint analyses requiring tissue harvesting (e.g., histology, immunohistochemistry, and RNA sequencing), a group size of 3–6 mice (n = 3–6) was used per group and time point, as specified in the respective figure legends. Mice were randomly assigned to experimental groups using a computer-generated randomization sequence after tumor implantation. The investigator responsible for measuring tumor bioluminescence and assessing survival endpoints was blinded to the group allocation throughout the study.

### 2.5 Bioluminescent imaging

G422^TN^-GBM cells were previously infected with a luciferase-expressing lentivirus and can therefore be used for *in vivo* tumor volume monitoring using the bioluminescent imaging (BLI) (Liu et al., 2023). BLI of intracranial G422^TN^-GBM tumors was performed using an animal *in vivo* optical imaging system (Spectral LagoX, United States) 10 min after a single intraperitoneal injection of 0.2 mL of sterile D-luciferin potassium salt (15 mg/mL in PBS, Cayman Chemical Company, 115144-35-9, United States). The region-of-interest (ROI) values measured using AMIView software (Spectral Instruments Imaging Company, United States) were used for statistical analysis of optical density values.

### 2.6 Cell proliferation assay

G422^TN^-GBM cells were seeded into 96-well plates at appropriate densities. Twenty-four hours later, ACT001 was added at concentrations of 10, 20, 30, 40, 50, 60, 70, 80, 90, or 100 μM. The viability and proliferation of cancer cells were measured at 0, 12, 24, and 48 h after drug incubation using the Cell Counting Kit-8 (CCK-8, G4103, ServiceBio, Wuhan, China), according to the manufacturer’s instructions.

### 2.7 Scratch-wound healing assay

As previously reported (Martinotti and Ranzato, 2020), cell migration ability was measured using the wound healing assay. In simple terms, a scratch was made on a six-well plate containing confluent cells using a pipette tip, and the wound edges were marked at 0 h and 24 h. The wound edge line was drawn by connecting the leading edge of at least 10 cells to the denuded area. The wound size was quantified using Image-Pro Plus (IPP) software (Media Cybernetics, United States) as the distance between the wound edges on opposite sides, and the average wound size represented at least five different regions per dish.

### 2.8 Hematoxylin–eosin staining and immunohistochemistry

Paraffin-embedded brain slices were used for hematoxylin–eosin (H&E) staining and immunohistochemistry (IHC) analysis, as previously described (Gao et al., 2025). In brief, 4-μm-thick brain slices were deparaffinized, rehydrated, blocked for endogenous peroxidase activity, subjected to antigen retrieval, blocked with 5% BSA, and incubated with primary and corresponding secondary antibodies (Polink-1 HRP DAB Detection System, PV-9001, ZSGB-BIO, Beijing, China), and colorimetric end products were generated by applying diaminobenzidine tetrahydrochloride. The following primary antibodies were used: anti-TNFα (1:500, A11534, ABclonal, Wuhan, China), anti-CXCL10 (1:200, PB0752, Boster, Wuhan, China), anti-PD-L1 (1:500, ab210931, Abcam, United Kingdom), anti-CD3 (1:2000, ab237721, Abcam, United Kingdom), anti-CD4 (1:2000, ab183685, Abcam, United Kingdom), anti-CD8 (1:2000, ab209975, Abcam, United Kingdom), and anti-KI67 (1:200, ab16667, Abcam, United Kingdom). Whole-brain images were obtained by scanning the brain sections at ×200 magnification using an automatic slice scanning system, SV120 (Olympus, Japan). Statistical analysis used data from six slices from six mouse brains per group. The data are expressed as the mean number of positive cells per square millimeter of microscopic field.

### 2.9 Terminal deoxynucleotidyl transferase dUTP nick-end labeling assay

Cell apoptosis levels in paraffin-embedded tissue sections were detected using an *In Situ* Cell Death Detection Kit (TMR red, 12156792910, Roche, Switzerland), following the manufacturer’s instructions. Six randomly selected fields per specimen were examined at ×200 magnification. The data are expressed as the mean number of apoptotic cells per square millimeter under microscopic observation.

### 2.10 RNA extraction and reverse transcription quantitative polymerase chain reaction

Total RNA was extracted from intracranial tumors using the Steady Pure Rapid RNA Extraction Kit (AG21023, Accurate Biology, Hunan, China), in accordance with the manufacturer’s protocol. Complementary DNA (cDNA) synthesis was performed using the Taq Pro Universal SYBR qPCR Master Mix (Q712-02, Vazyme, Nanjing, China). The expression of genes related to the following pathways was analyzed using the Quantagene q225 Real-Time PCR System: positive regulation of TNF signaling genes (*Laptm5*, *Casp1*, and *Adam17*), TNF production signature genes (*Ptafr*, *Ccl4*, and *Adam8*), phagocytosis activation genes (*Ano6*, *Arap1*, and *Ptx3*), T-cell activation genes (*Hsh2d*, *Tam1*, and *Rsad2*), apoptosis signaling genes (*Atf3*, *Il1b*, and *Sp100*), and immune checkpoint genes (*Pdl1* and *Pd1*). The primers of the above-mentioned genes are listed in Table 1(synthesized by Sangon Biotech, Shanghai, China).

**TABLE 1:** Primers sequences used for real-time PCR.

Gene (mouse)	Forward primer (5′ to 3′)	Reverse primer (3′ to 5′)
*β-actin*	AGC​CAT​GTA​CGT​AGC​CAT​CC	GAC​TCC​ATC​ACA​ATG​CCA​GT
*Rsad2*	GAA​CAG​CAC​TCA​GCC​CAC​AAC	GCA​GCA​CGA​AGG​ATG​TCT​TGG
*Tarm1*	CCT​CGG​ACC​CGT​AAA​GTT​TGC	GAG​TCT​CTG​GCT​GTC​ACA​TTC​TG
*Ccl4*	CTC​TGC​GTG​TCT​GCC​CTC​TC	GGT​GTA​AGA​GAA​ACA​GCA​GGA​AGT​G
*Sp100*	GTG​GTA​TGT​GGC​AAC​GCT​CAG	GAG​TGA​ATA​ACC​GCC​CTG​TCT​TG
*Atf3*	GAT​TTT​GCT​AAC​CTG​ACA​CCC​TTT​G	CTG​TTG​TTG​ACG​GTA​ACT​GAC​TCC
*Hsh2d*	TGG​CGT​GGA​AAG​GTG​GTA​AGG	GAA​AGG​TGG​TGG​TGG​AAG​GTA​TTC
*Adam8*	CCA​CTA​CCA​CGT​CTT​CTA​ATC​CAT​TG	TGA​CCA​TCG​CAG​CCA​CCA​G
*Ptafr*	GCA​CGG​TCT​TGG​CGG​TAT​TC	CTG​ATG​GAA​GTT​GGT​CTG​GTA​GC
*Ano6*	CAG​CCA​GTG​ACA​GGA​ACA​AG	GGC​ACA​GTC​AAA​GTC​GAT​GG
*Pdl1*	AAG​CCT​CAG​CAC​AGC​AAC​TTC​AG	TGT​AGT​CCG​CAC​CAC​CGT​AGC
*Pd1*	CAC​AGT​GTC​AGA​GGG​AGC​AAA​TG	GGC​GGT​TCC​AGT​TCA​GCA​TAA​G
*Arap1*	GAG​CCA​GAC​CTC​AAG​ACC​AA	TGG​CAT​AGA​TGC​CAC​CTT​CT
*Ptx3*	TGA​GAG​CAA​AGT​CAC​ACA​GCA	GGG​ACT​GGG​CTT​CAC​ATG​TA
*Laptm5*	TTT​CAA​CAT​CCG​AGT​CGC​CA	CCA​TCC​TGA​GGT​ACG​GCA​TC
*Casp1*	CGA​GGG​TTG​GAG​CTC​AAC​TT	AGA​AGT​CTT​GTG​CTC​TGG​GC
*Adam17*	CCA​GTA​CCT​GCA​GCT​CCA​A	GTG​ACT​GGG​TGG​TCT​GTG​AC
*Il1b*	GCA​ACT​GTT​CCT​GAA​CTC​AAC​T	ATC​TTT​TGG​GGT​CCG​TCA​ACT

### 2.11 Bulk RNA-sequencing data analysis

Fresh intracranial tumor tissues (>100 mg) were harvested from G422^TN^-GBM-bearing mice (n = 4 per group) on day 7 post-implantation following three consecutive days of treatment with either ACT001 or RT/TMZ for transcriptomic analysis. Total RNA was extracted, and cDNA libraries were constructed and sequenced on the Illumina HiSeq platform (PE150) by Novogene Bioinformatics Technology Co., Ltd. (Shanghai, China), yielding approximately 6 Gb of high-quality clean data per sample. For bulk RNA-seq data derived from different treatment groups (Ctrl, ACT001, RT/TMZ, and RT/TMZ/ACT001), raw reads were first processed using Trim Galore (version 0.6.10) to remove low-quality bases (Phred score <20) and adapter sequences, retaining reads with a minimum length of 50 nucleotides. The retained high-quality reads were then aligned to the mm10 mouse reference genome using the STAR aligner (version 2.7.10a) with default parameters (Dobin et al., 2013). Gene expression levels of all samples were quantified using RSEM (Sherman and Salzberg, 2020), which generated both raw counts and fragments per kilobase of transcript per million mapped reads (FPKM) values. Differential expression analysis was performed using the DESeq2 package (version 1.38.3) with the raw count matrix (Love et al., 2014). Differentially expressed genes (DEGs) were defined as those with an adjusted *p*-value (Benjamini–Hochberg correction) < 0.05 and an absolute log_2_ fold change ≥1 (Withanage et al., 2022). No batch correction was applied as all samples were processed in a single sequencing run. Bulk RNA sequencing data of our G422^TN^-GBM tumor can be downloaded from the National Center for Biotechnology Information (NCBI) under accession number PRJNA1298905.

### 2.12 Genomic data and clinical information

The American Cancer Genome Atlas (TCGA) and the Chinese Glioma Genome Atlas (CGGA) databases were used to collect glioma gene expression profiles and clinical information (Combes et al., 2022). The glioma dataset named GSE157779 (Hou et al., 2021) was downloaded from the Gene Expression Omnibus (GEO) (Chicco, 2022). FPKM was used to measure the gene expression levels, while read counts were downloaded for DEG analysis, along with clinical information for survival analysis (Combes et al., 2022).

### 2.13 Gene set and differential gene enrichment analysis

All pathway enrichment analyses were based on the Kyoto Encyclopedia of Genes and Genomes (KEGG) (Kanehisa et al., 2017) and Gene Ontology (GO) (Ashburner et al., 2000; The Gene Ontology Consortium, 2019) database resources. Gene set enrichment analysis (GSEA) software (version 4.3.2) was chosen to analyze the potential signaling pathways simultaneously. Gene sets of the TNF signaling pathway signature were downloaded from the Molecular Signatures Database (MSigDB) (Castanza et al., 2023).

### 2.14 Statistical analysis

Animal survival rates were analyzed using the Kaplan–Meier method, and comparisons were made using the log-rank (Mantel–Cox) test. Differences between the two unpaired groups were assessed using a two-tailed unpaired Student’s t-test. For comparisons among multiple groups, one-way ANOVA followed by Dunnett’s *post hoc* test was applied. Survival curves and body weight changes were statistically analyzed using GraphPad Prism 10. All data are presented as the mean ± SEM. *p*-value <0.05 was considered statistically significant.

## 3 Results

### 3.1 ACT001 significantly prolongs survival and suppresses tumor growth in G422^TN^-GBM mice

**FIGURE 1:**
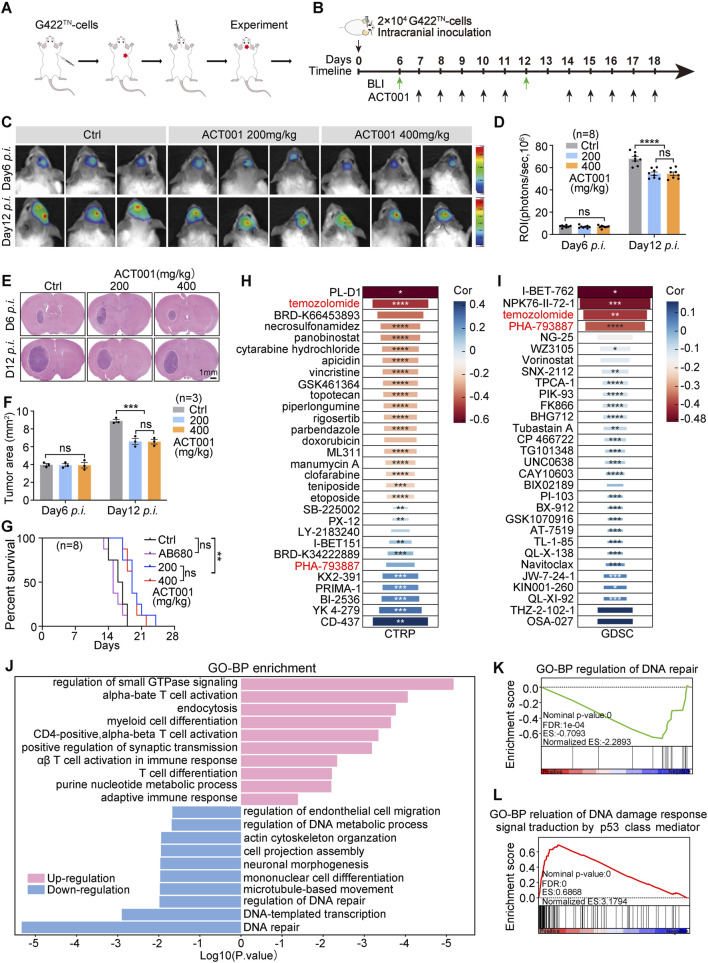
ACT001 prolongs the survival of G422^TN^-GBM mice. **(A)** Schematic diagram of the establishment of the orthotopic G422^TN^-GBM mouse model. **(B)** Schematic diagram depicting the BLI and therapeutic schedule of ACT001. ACT001 (200 mg/kg/day or 400 mg/kg/day) was administered p.o. via gavage. **(C,D)** Representative bioluminescent images **(C)** and statistical analysis of the ROI values **(D)** of tumor-bearing mice before and during treatment (days 6 and 12 post-implantation; n = 8 per group). **(E,F)** H&E staining images and statistical analysis of G422^TN^-GBM tumor in the Ctrl and ACT001 groups on days 6 and 12 *p.i.* (n = 3 per group). **(G)** The Kaplan–Meier survival curves of the ACT001, AB680, and Ctrl groups (n = 8/group). **(H,I)** Candidate drugs for ACT001 combination were screened using CTRP **(H)** and GDSC **(I)**, where red indicates strong correlation, blue shows weak correlation, negative values represent drug sensitization, and positive values denote drug resistance. **(J)** GO enrichment analysis of differentially expressed genes between ACT001-treated and control groups (data sourced from public databases). **(K,L)** Comparative GSEA analysis of the GSE157779 dataset showed significant suppression of DNA repair pathways **(K)** and concurrent activation of p53-mediated DNA damage response **(L)** in ACT001-treated mice. Data are presented as the mean ± SEM. Statistical significance for panels D and F was determined using the two-tailed unpaired Student’s t-test; survival differences in panel G were assessed using the log-rank (Mantel–Cox) test. Scale bar: 1 mm; *p < 0.05, **p < 0.01, ***p < 0.001, and ****p < 0.0001; ns, not significant.

To further investigate the therapeutic potential of ACT001 in glioma treatment, we utilized a highly stable and malignant mouse model called G422^TN^-GBM (Liu F. et al., 2020; Liu et al., 2023; Gao et al., 2025) (Figure 1A). Tumor-bearing mice were treated with ACT001 at doses of 200 mg/kg or 400 mg/kg (Figure 1B). BLI and H&E staining of brain tissues illustrated rapid tumor growth during days 6–12 post-implantation (p.i.), which was markedly attenuated by both doses of ACT001. No significant difference in antitumor efficacy was observed between the two dose groups (Figures 1C–F), and both treatments considerably extended the survival of G422^TN^-GBM-bearing mice, even compared to AB680 (CD73-targeted inhibitor) (Gao et al., 2025) (Figure 1G). Notably, both doses of ACT001 and AB680 monotherapy were well tolerated, with no considerable body-weight loss or other signs of toxicity observed compared to the vehicle control group (Supplementary Figure S3A). To evaluate the effects of ACT001 on G422^TN^-GBM and GL261 cells *in vitro*, we conducted cell viability assays using the CCK-8 method. The results indicated that both cell lines were sensitive to ACT001 treatment (Supplementary Figure S1A, B). In addition, scratch-wound healing assays revealed that incubation with ACT001 markedly suppressed the migratory ability of G422^TN^-GBM and GL261 cells (Supplementary Figure S1C, E). Collectively, these *in vitro* findings demonstrated that ACT001 could effectively inhibit the proliferation and migration of GBM cells.

The Clinical Trials Research Platform (CTRP) (https://portals.broadinstitute.org/ctrp/) and the Genomics of Drug Sensitivity in Cancer (GDSC) (https://www.cancerrxgene.org/) databases simultaneously suggested that TMZ and PHA-793887 (CDK inhibitor) might increase the ACT001 drug sensitivity level (Figures 1H,I). Meanwhile, GO Biological Process (GO-BP) enrichment analysis of DEGs after ACT001 treatment revealed significant downregulation in DNA repair (Figures 1J,K), in contrast to the upregulation in DNA damage (Figure 1L). This body of evidence demonstrated that ACT001 can be used in combination with TMZ to exert a synergistic effect and further inhibit GBM progression.

### 3.2 ACT001 synergizes with RT/TMZ to enhance therapeutic efficacy and achieve long-term survival in G422^TN^-GBM mice

**FIGURE 2:**
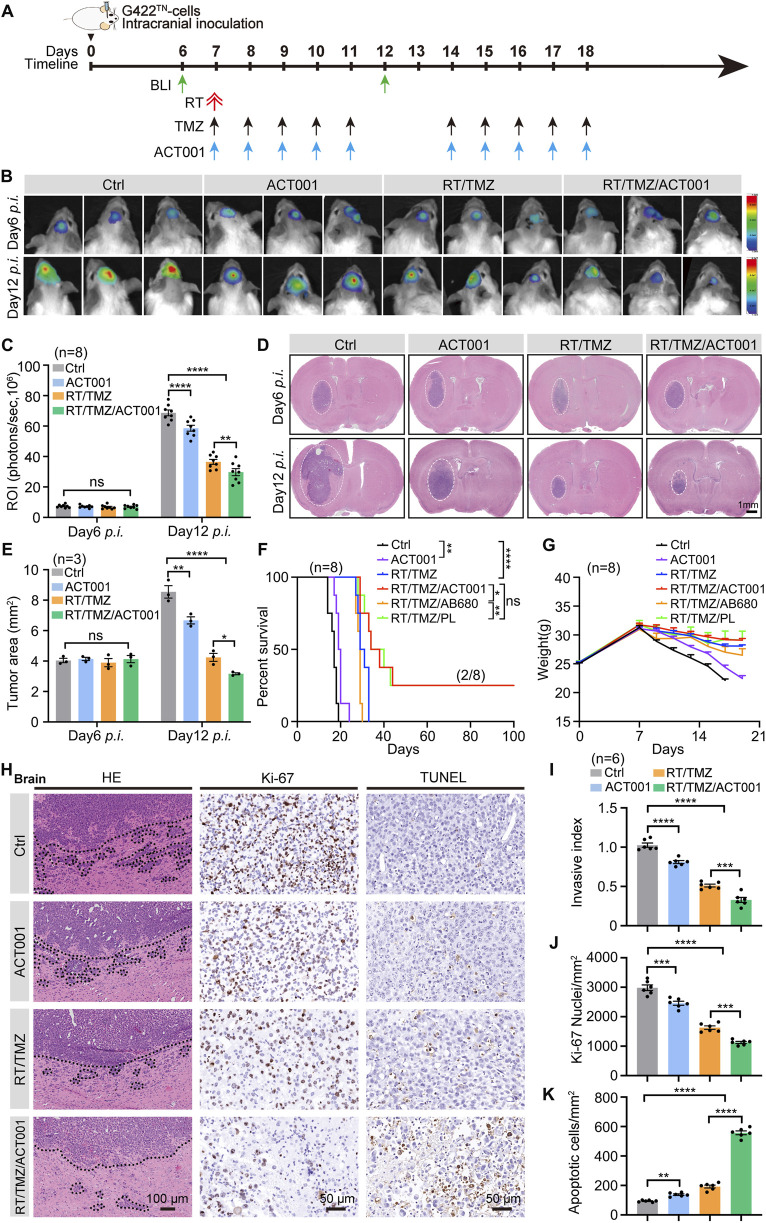
ACT001 significantly enhances the therapeutic efficacy of RT/TMZ in G422^TN^-GBM mice. **(A)** Therapeutic schedule of ACT001, RT/TMZ, or combined regimen started on day 7 p.i. RT, a single dose of 10 Gy whole-brain irradiation (WBI); ACT001 (200 mg/kg/day) was administered p.o. via gavage. TMZ (50 mg/kg/day) was administered p.o. via gavage. **(B,C)** Representative bioluminescent images **(B)** and statistical analysis **(C)** of the ROI values of tumor-bearing mice on days 6 and 12 post-implantation (n = 8 per group). **(D,E)** H&E staining images **(D)** and statistical analysis **(E)** of tumor in the Ctrl, ACT001, RT/TMZ, and RT/TMZ/ACT001 groups on days 6 and 12 post-implantation (n = 3 per group). **(F,G)** Kaplan–Meier survival curves **(F)** and body-weight change curves for all treatment groups **(G)** of G422^TN^-GBM-bearing mice treated with the following regimens: vehicle control (Ctrl), ACT001 monotherapy, RT/TMZ alone, RT/TMZ combined with ACT001, RT/TMZ combined with AB680, or RT/TMZ combined with PL (n = 8 per group). The delayed and more gradual body-weight decrease in treatment groups compared to the control is consistent with the reduced tumor burden. **(H–K)** H&E staining **(H)**, TUNEL assay **(I)**, Ki-67 immunohistochemistry **(J)**, and corresponding quantitative analyses **(K)** of G422^TN^ tumor in the indicated groups on day 9 post-implantation (n = 3 per group). Data are presented as the mean ± SEM. Statistical comparisons for panels C, E, and K were performed using the one-way ANOVA, followed by Dunnett’s *post hoc* test; survival differences (panel F) were analyzed using the log-rank test. Scale bars: 1 mm (H&E overview), 100 µm (H&E insets), and 50 µm (TUNEL, Ki-67); *p < 0.05, **p < 0.01, ***p < 0.001, and ****p < 0.0001; ns, not significant.

Having established the monotherapy efficacy of ACT001, we investigated its combinatorial potential with RT/TMZ (Figure 2A). At day 12 p.i., tumor suppression was more pronounced in the RT/TMZ group than in the ACT001 monotherapy, while the RT/TMZ/ACT001 combination demonstrated superior efficacy in reducing the G422^TN^-GBM tumor burden, which was confirmed by both BLI and tumor area variation (Figures 2B–E). Survival analysis revealed that RT/TMZ treatment yielded a median survival of 30 days, whereas the combination therapy extended median survival by 8 days and produced 20% (2/8) long-term survival (LTS) mice (Figure 2F). For this study, “cure” was operationally defined as survival exceeding 100 days without signs of tumor recurrence, a threshold chosen based on the following: (1) the median survival of control mice in this model being <30 days, making 100 days a stringent benchmark; and (2) alignment with previous studies using this model to define durable remission (Liu F. et al., 2020; Liu et al., 2023). Consistent with the favorable safety profile of ACT001 monotherapy (see Section 3.1), the RT/TMZ/ACT001 combination regimen was also well tolerated, with no treatment-related toxicity observed (Figure 2G). Compared to AB680 and PL (Liu et al., 2023), which had been combined with RT/TMZ in the same G422^TN^-GBM model, RT/TMZ/ACT001 demonstrated a considerable survival benefit over RT/TMZ/AB680 and achieved a similar survival extension to RT/TMZ/PL (Figure 2F). Furthermore, H&E staining and IHC results verified that RT/TMZ/ACT001 significantly reduced G422^TN^ cell invasion (invasive index) and proliferation (Ki-67) while promoting tumor cell apoptosis (TUNEL) in the tumor parenchyma (Figures 2H–K). These findings support that ACT001 potently enhances the therapeutic efficacy of RT/TMZ.

### 3.3 ACT001 activates the TNF signaling pathway to enhance the antitumor effects of RT/TMZ

**FIGURE 3:**
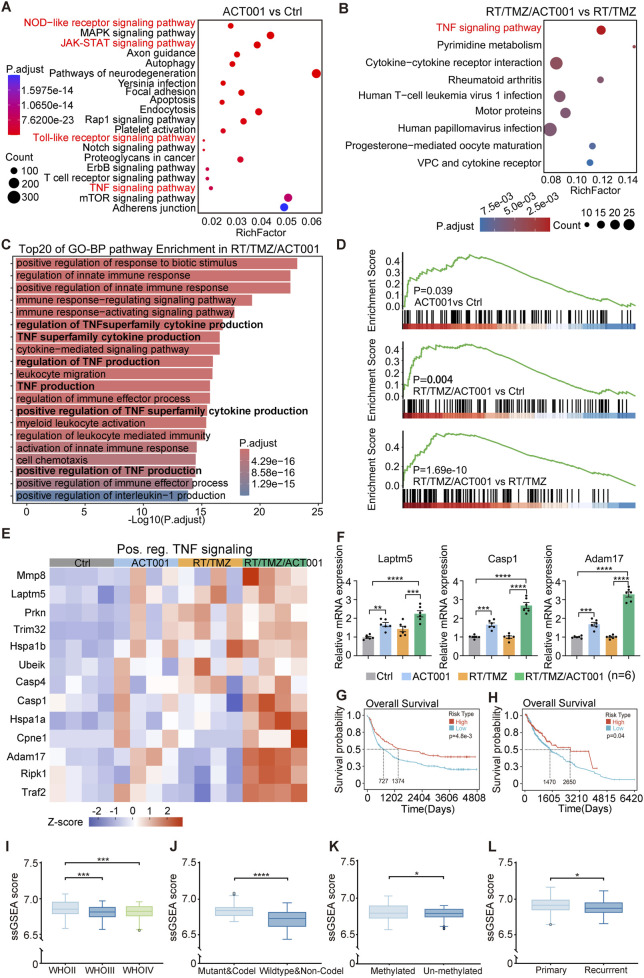
ACT001 activates the TNF signaling pathway to enhance the antitumor effects of RT/TMZ. **(A)** KEGG enrichment analysis comparing all upregulated genes in the ACT001 with the control. **(B)** KEGG enrichment analysis comparing all upregulated genes in RT/TMZ/ACT001 with RT/TMZ. **(C)** GO pathway analysis comparing the TNF signaling pathway in RT/TMZ/ACT001 with the control. **(D)** GSEA of the TNF signaling pathway in ACT001 (compared to the control) and RT/TMZ/ACT001 (compared to the control and RT/TMZ).The enrichment of the TNF signaling pathway was significant after FDR correction (q < 0.05). **(E)** The heatmap visualizes upregulated TNF signaling-related gene expression in each group. **(F)** RT-qPCR showing the mRNA expression levels of some differentially expressed genes in the TNF signaling pathway (*Laptm5*, *Casp1*, and *Adam17*). **(G,H)** The Kaplan–Meier analysis of patients’ OS between the high-TNF and the low-TNF score expression groups in CGGA325 **(G)** and TCGA–LGG and GBM **(H)** datasets. **(I**–**L)** Boxplot illustrating the TNF signature scores across different histologies **(I)**, IDH mutation status **(J)**, MGMT promoter methylation status **(K)**, and tumor recurrence status **(L)** in the CGGA325 glioma dataset. Statistical significance for panel F was determined using the one-way ANOVA with Dunnett’s *post hoc* test; survival differences **(G,H)** were assessed using the log-rank test; group comparisons in panels I–L were performed using the Kruskal–Wallis test or Mann–Whitney U-test as appropriate. *p < 0.05, **p < 0.01, ***p < 0.001, and ****p < 0.0001; ns, not significant.

To explore the mechanisms underlying the synergistic effects of ACT001 in combination with RT/TMZ, we conducted bulk RNA sequencing analysis. DEGs between each treatment group were used for enrichment analysis. KEGG enrichment analysis comparing the ACT001 group with the control, along with RT/TMZ/ACT001 with RT/TMZ, revealed significant enrichment of the TNF signaling pathway (Figures 3A,B). In particular, GO-BP enrichment analysis of highly expressed genes in the RT/TMZ/ACT001 group showed that the TNF signaling pathway played an important role in the antineoplastic mechanism (Figure 3C), with GSEA focusing on the upward trend of the TNF signaling pathway (Figure 3D). Among the DEGs, genes involved in the positive regulation of TNF signaling were selected for validation by RT-qPCR analysis; this confirmed the DEGs identified by the bioinformatics analysis and was consistent with the trends observed in our RNA-seq data (Figures 3E,F). These results supported that ACT001 enhances the antitumor effects of RT/TMZ by upregulating the TNF signaling pathway.

We further generated a TNF signaling score using a gene set (Table 1) to evaluate its clinical correlations. In the TCGA and CGGA databases, we found that a higher signature score of TNF signaling was significantly associated with longer overall survival (OS) in patients with glioma (Figures 3G,H). In addition, clinical information, WHO pathological grade, IDH mutation, 1p19q codeletion status, MGMT methylation, and recurrence status were used as indices, indicating that a higher TNF signaling signature score corresponded to a lower degree of malignancy (Figures 3I–L), which can serve as a favorable prognostic factor.

### 3.4 RT/TMZ/ACT001 combination results in the activation of antitumor signaling pathways

**FIGURE 4:**
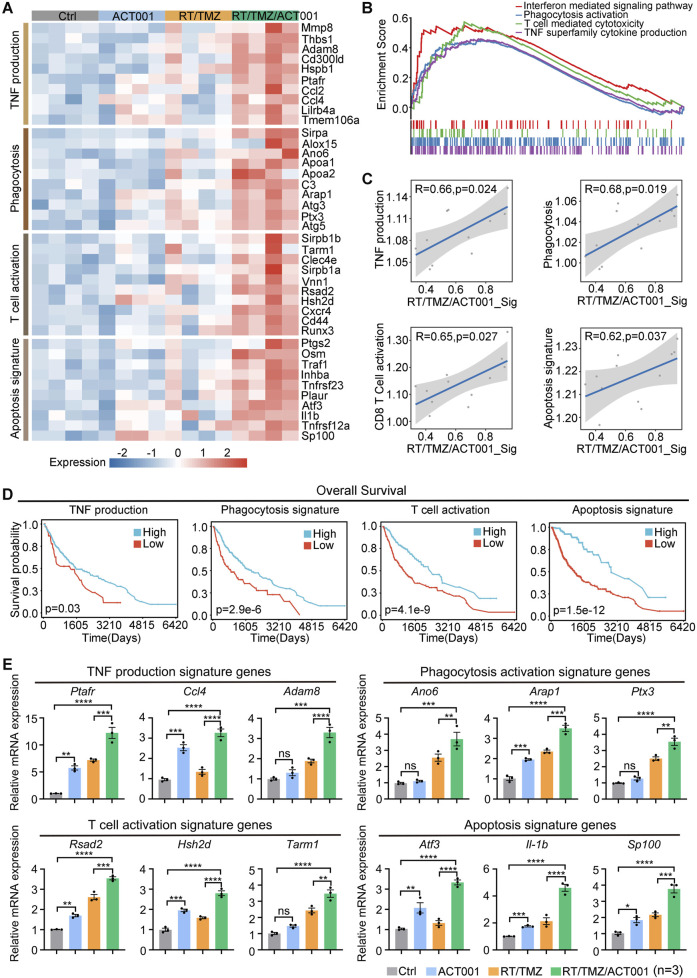
RT/TMZ/ACT001 in combination generates the activation of anti-tumor signaling pathways. **(A)** Heatmap showing the expression of genes related to tumor necrosis factor production, phagocytosis, T-cell activation, and apoptosis signaling in different treatment groups. **(B)** Enrichment of all TNF signaling pathway genes in the RT/TMZ/ACT001 compared to the control. **(C)** Correlation analysis of TNF production, phagocytosis, CD8^+^ T-cell activation, and apoptotic signaling. **(D)** The Kaplan–Meier analysis of patients’ OS grouping based on high and low TNF production, phagocytosis signature, T-cell activation, and apoptotic signature scores in the TCGA–LGG and GBM dataset. **(E)** RT-qPCR showing the mRNA expression of genes related to tumor necrosis factor production (*Ptafr*, *Ccl4*, and *Adam8*), phagocytosis activation (*Ano6*, *Arap1*, and *Ptx3*), T-cell activation (*Rsad2*, *Hsh2d*, and *Tam1*), and apoptosis signaling (*Atf3*, *Il1b*, and *Sp100*) (n = 3 per group). Data are presented as the mean ± SEM. Statistical significance for panel E was determined using the one-way ANOVA, followed by Dunnett’s *post hoc* test. *p < 0.05, **p < 0.01, ***p < 0.001, and ****p < 0.0001; ns, not significant.

Based on the finding that the TNF signaling pathway was activated, we further revealed four key biological processes related to the TNF signaling pathway through gene expression level analysis: tumor necrosis factor production, phagocytosis, T-cell activation, and apoptosis signaling, as well as in GSEA analysis (Figures 4A,B). In the TCGA database, among patients with LGG and GBM, higher signature scores of these four pathways indicated better prognosis (Figure 4C). More importantly, the combination group showed significant advantages in promoting the above-mentioned four biological processes (Figure 4D). To verify these findings, we selected representative genes from each biological process for mRNA level validation, and the results showed that the expression of these genes was highest in the combination treatment group (Figure 4E), consistent with the previous conclusion. Together, these results support that RT/TMZ and ACT001 synergistically induced G422^TN^-tumor gene reprogramming toward inflammatory immune responses.

### 3.5 ACT001 enhances the antitumor immune efficacy of RT/TMZ by promoting the TNF–CXCL10–CD8^+^ T-cell axis

**FIGURE 5:**
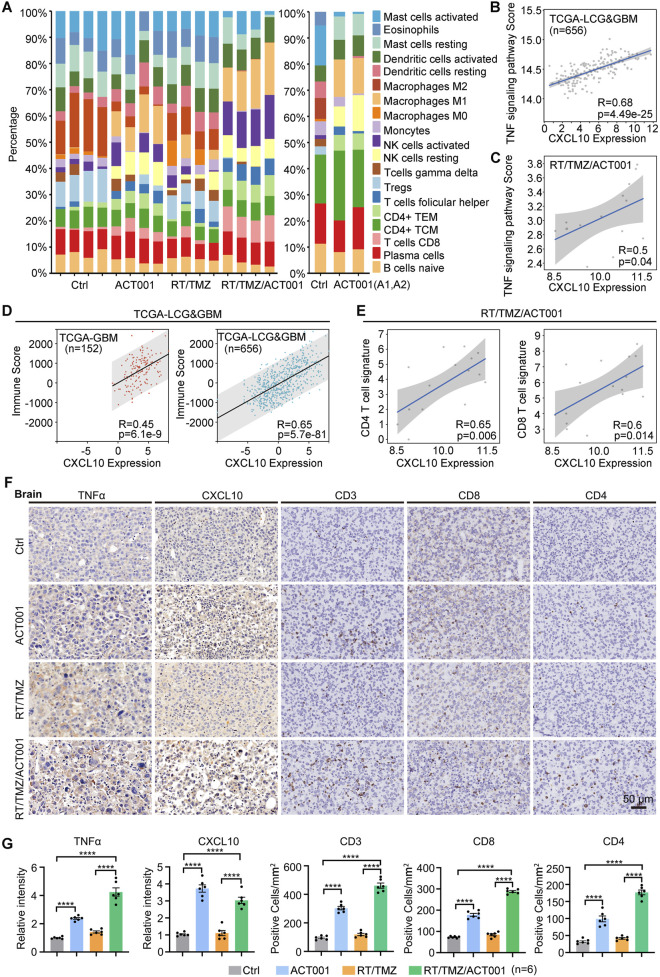
ACT001 enhances the antitumor immune efficacy of RT/TMZ by promoting the TNFα–CXCL10–CD8^+^ T-cell axis. **(A)** CIBERSORT analysis of immune-cell proportions derived from RNA-seq data (left) and the GSE157779 dataset (right). **(B,C)** Correlation analysis between CXCL10 expression and TNF-signature scores in the TCGA–LCG and GBM cohort **(B)** and in the RT/TMZ/ACT001 RNA-seq data **(C)**. **(D)** Correlation analysis between CXCL10 expression and immune score in the TCGA–GBM and the TCGA–LCG and GBM cohorts. **(E)** Correlation analysis between CXCL10 expression and CD4^+^ or CD8^+^ T-cell signature scores in the RT/TMZ/ACT001 RNA-seq dataset. **(F,G)** IHC staining (TNFα, CXCL10, CD3, CD8, and CD4) and statistical analysis of G422^TN^-GBM tumors from the indicated groups on day 9 post-implantation (n = 6 per group). Data are presented as the mean ± SEM. Statistical comparisons for panel G were performed using the one-way ANOVA, followed by Dunnett’s *post hoc* test. Scale bar: 50 μm; ****p < 0.0001.

To further evaluate the immune microenvironment reprogramming induced by the combined treatment, we used CIBERSORT to analyze immune cell proportions based on RNA-seq and GSE157779 data (Hou et al., 2021). CIBERSORT analysis of immune cell proportions using both our RNA-seq data and the independent GSE157779 dataset revealed that the RT/TMZ/ACT001 combination significantly increased the infiltration of NK cells, CD4^+^ T cells, and CD8^+^ T cells (Figure 5A; Supplementary Figure S2A). Notably, analysis of the GSE157779 dataset indicated that ACT001 monotherapy itself substantially reshaped the immune landscape, and this immunomodulatory effect was further enhanced when combined with RT/TMZ. The relevance of CXCL10 to the TNF signaling pathway, observed in TCGA–LGG and GBM databases and our RNA-seq data, together with the notable enhancement of TNFα expression and pathway activity upon treatment (Figures 5B,C; Supplementary Figure S2B, C), collectively suggested that the RT/TMZ/ACT001 combination therapy reshapes the immunosuppressive tumor microenvironment. This remodeling effect was likely mediated by the activation of the TNF signaling pathway, which modulated the expression profile of immune cells. Additionally, immune scores associated with the CXCL10 expression level in TCGA–LGG and GBM demonstrated a positive correlation, which was consistent with the association between CD4^+^ and CD8^+^ T-cell signature scores and CXCL10 expression levels in the sequencing data of the RT/TMZ/ACT001 group (Figures 5D,E). The tissue-level validation results were highly consistent with the aforementioned findings: the expression levels of TNFα, CXCL10, CD3^+^ T cell, CD8^+^ T cell, and CD4^+^ T cell were considerably higher in the ACT001 treatment group and the RT/TMZ/ACT001 combination treatment group than in the control group and the RT/TMZ treatment group (Figures 5F,G), especially CD8^+^ T cell, which was considered to have played the main role in tumor killing function. These results collectively revealed that ACT001 enhanced the antitumor effects of RT/TMZ by upregulating TNF signaling to promote CXCL10 production, which, in turn, increased T-cell infiltration, forming TNF–CXCL10–CD8^+^ T-cell axis (Figure 7).

### 3.6 TNF signaling mediates ACT001’s antitumor efficacy, while PD1 blockade fails to provide additional therapeutic benefit in G422^TN^-GBM

**FIGURE 6:**
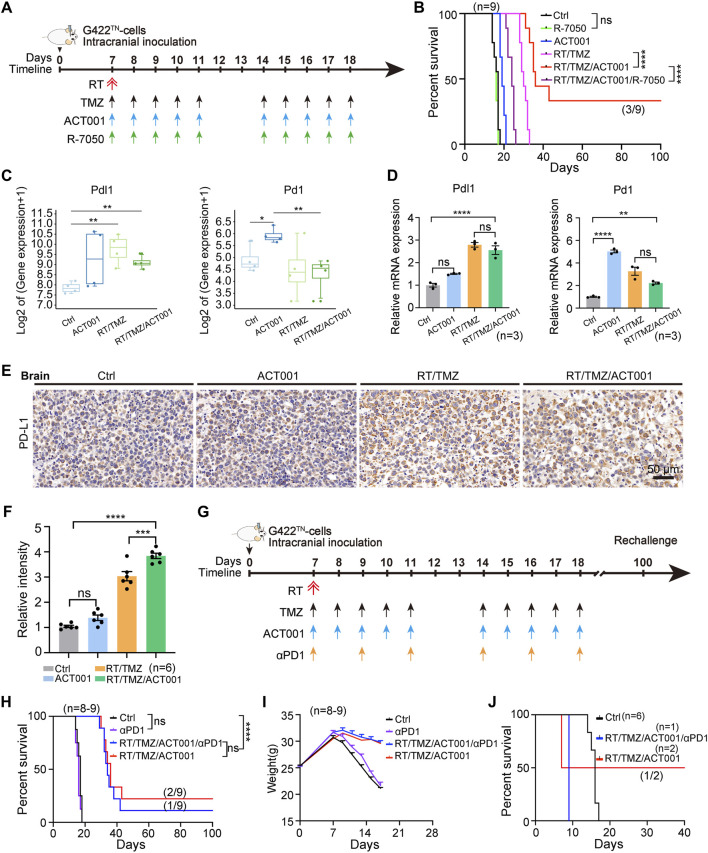
TNF signaling mediates ACT001’s antitumor efficacy, while PD1 blockade fails to provide additional therapeutic benefit in G422^TN^-GBM mice. **(A)** Therapeutic schedule of ACT001, R-7050, RT/TMZ, or their combinations. ACT001 (200 mg/kg/day), R-7050 (5 mg/kg/day), and TMZ (50 mg/kg/day) were administered orally. **(B)** Kaplan–Meier survival curves of G422^TN^ mice treated with the indicated regimens. **(C)** Expressions of PD-L1 and PD-1 in different treatment groups**. (D)** RT-qPCR analysis of mRNA expression of the immune-checkpoint molecules PD-L1 and PD-1 (n = 3 per group). **(E,F)** IHC staining **(E)** and statistical analysis **(F)** of PD-L1 in tumors from the indicated groups (n = 6 per group). **(G)** Schematic diagram of the treatment regimen. The dosing schedules for ACT001 and RT/TMZ were the same as before; αPD1 was administered intraperitoneally on day 7 post-implantation (initial dose 400 µg/mouse, followed by 200 µg/mouse every other day for a total of six doses). **(H,I)** Kaplan–Meier survival curves **(H)** and body-weight change curves **(I)** of G422^TN^-bearing mice treated with αPD-1, RT/TMZ/ACT001, or their combination (n = 8–9 per group). **(J)** Survival curves of tumor-bearing mice after tumor rechallenge in the control group (n = 6), the RT/TMZ/ACT001 group (n = 1), and the RT/TMZ/ACT001/αPD1 group (n = 2). Data are presented as the mean ± SEM. Statistical comparisons for panels D and F were performed using the one-way ANOVA with Dunnett’s *post hoc* test; survival differences (panels B, H, and J) were analyzed using the log-rank test. Scale bar: 50 μm; **p < 0.01, ***p < 0.001, and ****p < 0.0001; ns, not significant.

Then, we needed to verify whether using the inhibitors of the TNF signaling pathway (R-7050) in the combination therapy group could reverse the therapeutic effect, thereby further confirming that the TNF signaling pathway was the primary mechanism of action for the antitumor efficacy of ACT001. Although R-7050 alone did not affect survival and was well tolerated (no toxicity observed; Supplementary Figure S3B), R-7050 completely abolished the synergistic efficacy of RT/TMZ/ACT001, with a median survival time less than that of the RT/TMZ group (Figures 6A,B). This result suggested that the TNF signaling pathway was a key therapeutic mechanism underlying the action of RT/TMZ/ACT001. The PD-1 inhibitor remained a commonly used immunotherapy in clinical practice. Both expression levels of PD-L1 and PD-1 increased in the RT/TMZ/ACT001 group in our RNA sequencing data, compared with the Ctrl group, in accordance with quantitative RT-qPCR and IHC analyses (Figures 6C–F). We examined the efficacy of αPD1 in combination with RT/TMZ/ACT001 therapies started on day 7 p.i. in orthotopic G422^TN^-GBM mice (Figure 6G). αPD1 monotherapy did not contribute to animal survival, while αPD1, along with RT/TMZ/ACT001, prolonged OS, with one (1/9) animal achieving LTS, showing less efficacy than the RT/TMZ/ACT001 group, and two (2/9) animals achieving LTS, but without statistical significance (RT/TMZ/ACT001 vs. RT/TMZ/ACT001/αPD1, *p* > 0.05) (Figures 6H,I). All animals achieving long-term survival (LTS) from the RT/TMZ/ACT001 group (two mice) and the RT/TMZ/ACT001/αPD1 group (one mouse) were subjected to the tumor-rechallenge assay (Figure 6J). Only one (1/2) from the RT/TMZ/ACT001 group survived the rechallenge >100 days, achieving what we defined as “immune cure” (i.e., survival past the primary cure threshold with confirmed resistance to tumor rechallenge). Due to the limited number of LTS mice available for rechallenge (n = 2 for RT/TMZ/ACT001; n = 1 for RT/TMZ/ACT001/αPD1), the rechallenge results, while biologically informative in demonstrating complete immune protection in one instance, are not suitable for formal statistical comparison between groups. These results demonstrated that RT/TMZ/ACT001 therapy can achieve cure and immune cure in this highly refractory GBM model. Under the experimental conditions tested, the addition of αPD1 to this regimen did not provide a statistically significant additional survival benefit.

## 4 Discussion

**FIGURE 7:**
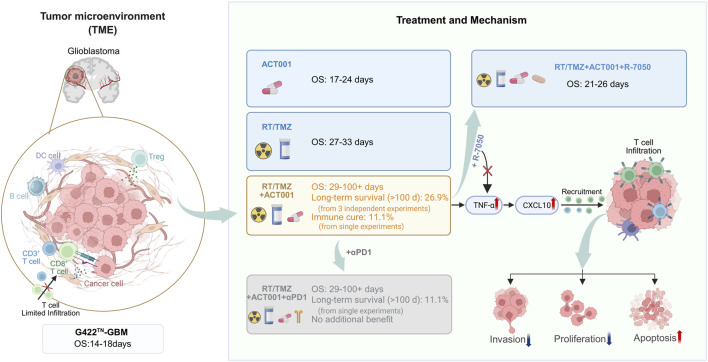
Comparative schematic diagram of treatment effects on the glioblastoma microenvironment and survival outcomes. The illustration contrasts the tumor immune landscape and key efficacy metrics (median overall survival [OS] extension, long-term survival [LTS], and immune-cure rates) following monotherapies (RT/TMZ or ACT001) versus the RT/TMZ/ACT001 combination, which activates the TNFα–CXCL10–CD8+T-cell axis (created with BioRender).

In this study, we demonstrate that ACT001 synergizes with RT/TMZ to achieve long-term survival and immune cure in a highly refractory orthotopic GBM model. In contrast to the mechanisms targeted by piperlongumine (PL, which induces oxidative stress) or the CD73 inhibitor AB680 (which modulates purinergic signaling) in this model (Liu et al., 2023; Gao et al., 2025), we identify the activation of the TNFα–CXCL10–CD8^+^ T-cell axis as a novel and effective immunomodulatory strategy for combining with standard chemoradiotherapy. Our findings reveal that ACT001 activates the TNF signaling pathway, leading to upregulation of TNFα and CXCL10 expression, which, in turn, promotes the infiltration of CD3^+^ and CD8^+^ T cells into the tumor microenvironment. Crucially, we mechanistically validated the pivotal role of TNF signaling in this process as pharmacological blockade of TNF markedly attenuated the anti-tumor effects of RT/TMZ/ACT001 (Figure 7).

TNF signaling is a well-established master regulator of anti-tumor immunity (Webster and Vucic, 2020; Alim et al., 2024), capable of inducing tumor cell death, disrupting tumor vasculature, and sensitizing cells to DNA-damaging therapies (Gupta et al., 2013; Gkretsi et al., 2017; Chang et al., 2019; Jo et al., 2020)—the latter being particularly relevant in the context of our chemoradiotherapy regimen. Beyond these canonical roles, we reveal a distinct immunomodulatory axis in GBM: TNF pathway activation driven by ACT001 particularly upregulates the T-cell chemoattractant CXCL10 (He et al., 2024). Our data directly link this to enhanced CD3^+^ and CD8^+^ T-cell infiltration. Given that insufficient T-cell infiltration is a major therapeutic bottleneck in GBM (Chongsathidkiet et al., 2018; Ye et al., 2023; Qiu et al., 2025), this mechanism directly addresses a central clinical challenge and underlies the observed synergistic cure.

The essential role of TNF signaling was further confirmed through loss-of-function experiments using TNF blockade. Upon TNF inhibition, the therapeutic efficacy of RT/TMZ/ACT001 was completely diminished as the survival advantage disappeared, strongly suggesting that baseline TNF activity is a fundamental requirement for treatment response. This observation has important clinical implications as it may explain intrinsic resistance to standard therapy in certain patient subgroups (Kalbasi and Ribas, 2020; Adhikari et al., 2022) and indicates that TNF signaling expression levels could serve as a predictive biomarker for treatment stratification (Wu et al., 2025). The role of the TNF signaling pathway in inhibiting angiogenesis and activating other types of immune cell types still requires further investigation and clarification (Hijdra et al., 2012).

Importantly, the RT/TMZ/ACT001 regimen was well tolerated in our studies, with no observed treatment-related toxicity, aligning with the favorable safety profile reported for ACT001 in early-phase clinical trials (e.g., ChiCTR-OIC-17013604). The dose used (200 mg/kg) was effective in our refractory model and translates to a clinically relevant range, providing a rational basis for future translational studies.

The complexity of combining ACT001 with immune checkpoint inhibitors warrants discussion. Although the dual therapy of ACT001 with RT/TMZ yielded favorable therapeutic outcomes, extending this combination to include anti-PD-1 therapy as a triple regimen failed to provide additional survival benefits. This unexpected result may stem from several key factors: 1) temporal dynamics of immune activation: Treatment sequencing may be critical (Zhu et al., 2022; Mortezaee and Majidpoor, 2023). Although ACT001 promotes T-cell infiltration, concurrent administration of anti-PD-1 may be insufficient to overcome the profoundly immunosuppressive GBM microenvironment. Staggered dosing regimens warrant investigation. 2) Compensatory immune checkpoint activation (Toor et al., 2020; Jiang et al., 2025): TNF-mediated immune activation induced by ACT001 may trigger upregulation of alternative immune checkpoints (e.g., LAG-3 and TIM-3) that remain unaffected by anti-PD-1 monotherapy. 3) Dual nature of TNF signaling (Gonzalez Caldito, 2023; Renz et al., 2024): Although TNF signaling is essential for immune activation, excessive TNF signaling may paradoxically foster immunosuppressive mechanisms—through NF-κB activation or recruitment of regulatory T-cell populations (Nishikawa and Koyama, 2021). These findings underscore both the therapeutic potential and inherent limitations of this immunomodulatory strategy. It is important to acknowledge the limitations of this particular experiment, including its modest sample size, the fixed concurrent dosing schedule employed, and the possibility that alternative treatment sequences might yield different outcomes. Therefore, our results indicate that the specific triple-combination regimen tested here does not synergize, but they do not preclude the potential utility of PD-1 blockade in alternative schedules or patient subsets defined by specific immune contexts.

### 4.1 Limitations and future perspectives

Although our study establishes the TNFα–CXCL10–CD8^+^ T-cell axis as a promising mechanism, several limitations should be noted. First, our conclusions are drawn primarily from a single, albeit highly refractory, transgenic GBM model. The generalizability of this immunomodulatory strategy to other GBM subtypes warrants further investigation. Second, although our multi-omics and pharmacological inhibition data strongly implicate this axis, definitive proof of causality through CXCL10 neutralization or CD8^+^ T-cell depletion experiments is lacking and represents an important direction for future mechanistic studies. Third, regarding toxicity profiling, we have addressed this point comprehensively in the *Results* (Sections 3.1 and 3.2), confirming that the regimens were well tolerated. These limitations highlight specific avenues for future research to translate these promising preclinical findings.

In conclusion, this study establishes ACT001 as an effective enhancer of RT/TMZ therapy in GBM via TNFα-mediated immune activation. Although the concurrent addition of PD-1 blockade to the RT/TMZ/ACT001 regimen did not enhance efficacy in this model, these results provide crucial insights into the biological complexities of immunomodulation in GBM and will help inform the rational design of future combination therapies. This study highlights the immense promise of targeted immunomodulation and the substantial challenges inherent in translating these approaches into meaningful clinical benefits for patients with GBM.

## Data Availability

The datasets presented in this study can be found in online repositories. The names of the repository/repositories and accession number(s) can be found at: https://www.ncbi.nlm.nih.gov/, PRJNA1298905.
