# Supplementary material for: ACT001 synergizes with temozolomide-based chemoradiotherapy to cure refractory glioblastoma by targeting TNF-CXCL10-CD8+ T-cell immunity
Source: Front Pharmacol. 2026 Feb 12;17:1745656. doi: 10.3389/fphar.2026.1745656 (PMC12935934; doi:10.3389/fphar.2026.1745656)
Supplement: Supplementary file 1 [file Supplementaryfile1.docx]

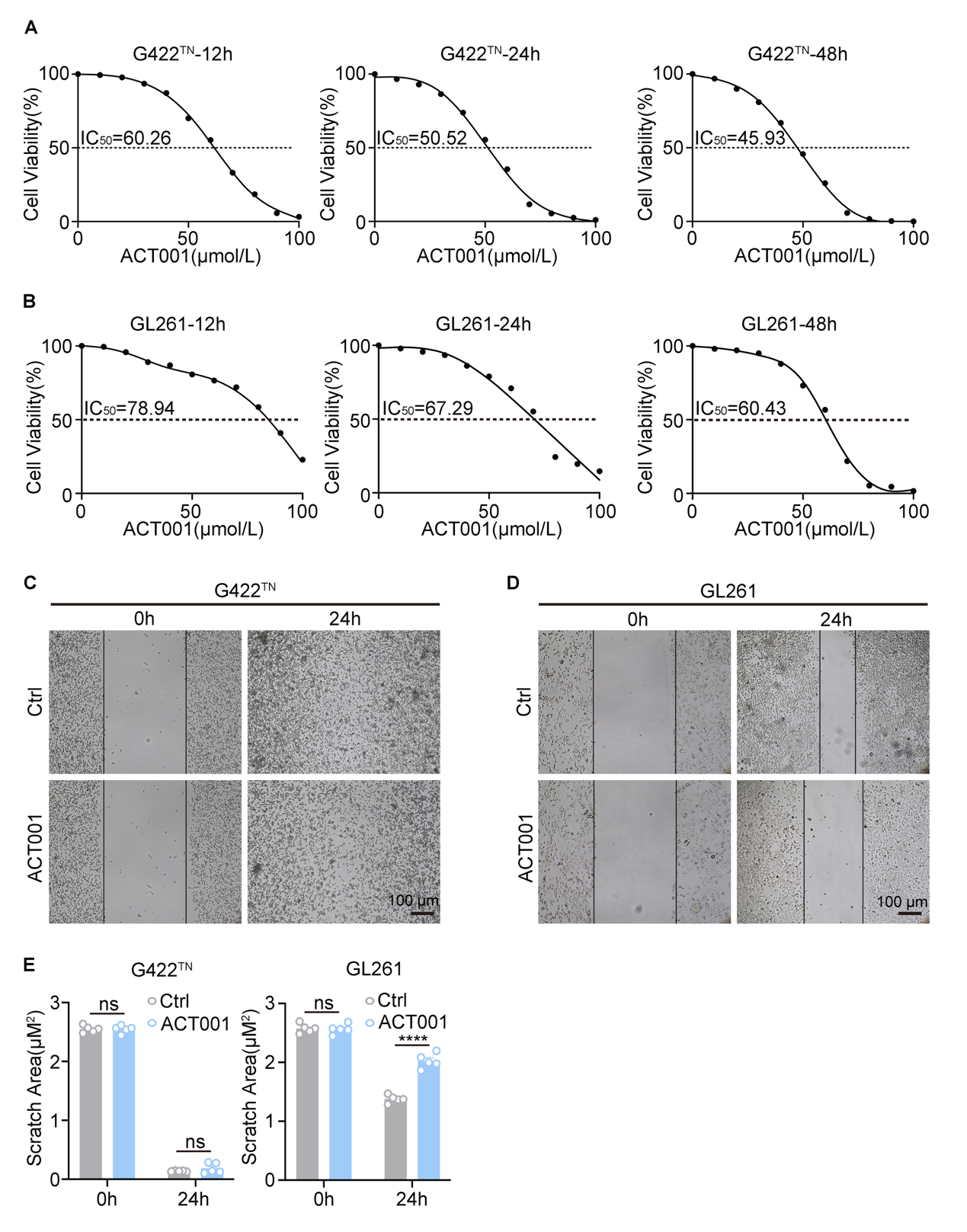
 **Fig. S1.** ACT001 suppresses GBM cell functions in vitro. **(A-B)** Time and dosage-dependent cell viability of ACT001-treated G422^TN^-GBM and GL261 cells (CCK-8 assay) at 12 h, 24 h, and 48 h (n = 3 independent experiments ). **(C-E)** Scratch wound assay and statistical analysis of G422^TN^-GBM and GL261 cells migration pre- and post-ACT001 treatment. Scale bar: 100 μm; ****P < 0.0001; ns, not significant.

**
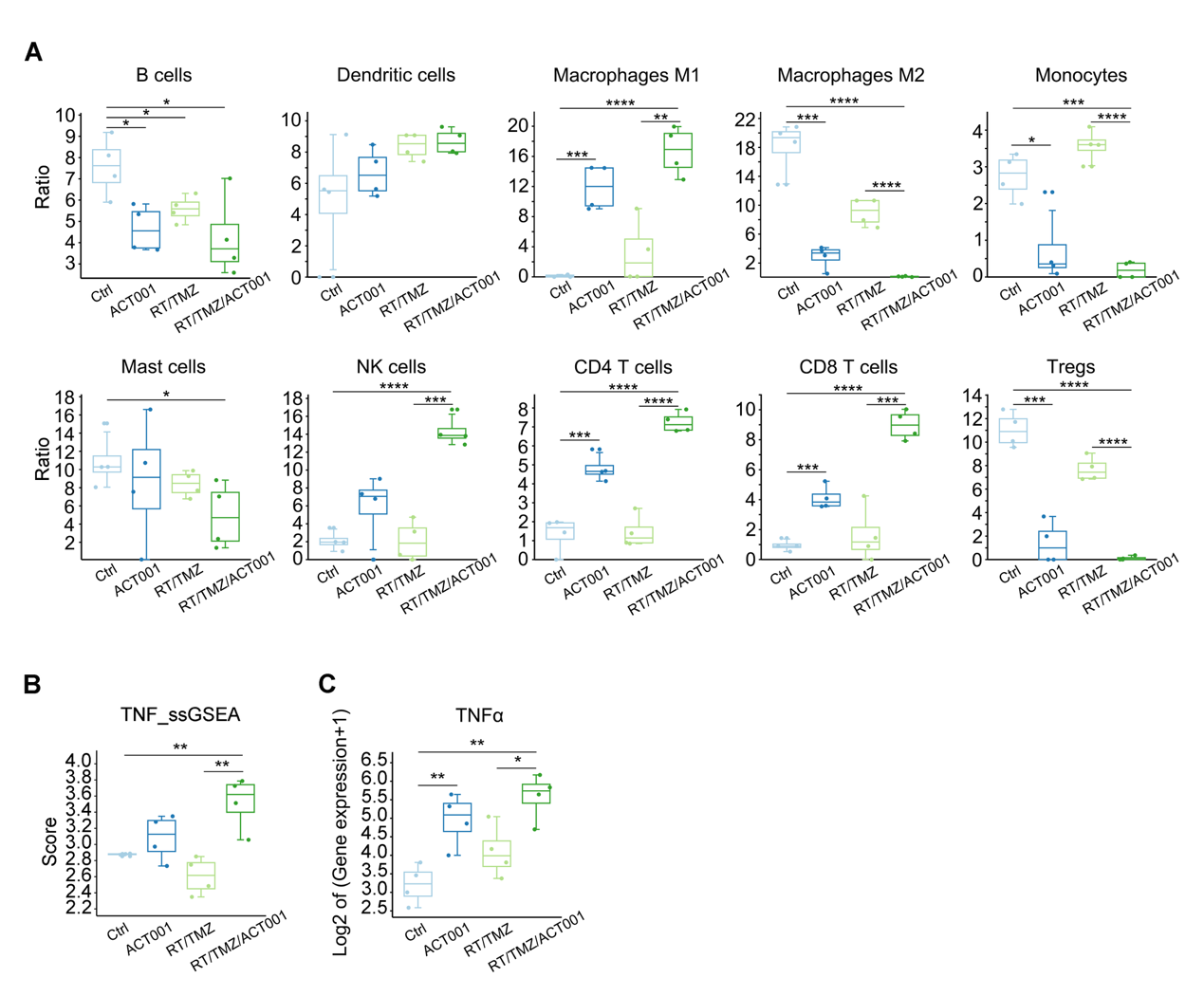
Fig. S2. Immune microenvironment characterization and TNF signaling pathway activation analysis (A)** Immune cell proportion analysis in each group. **(B)** ssGSEA analysis TNF signaling pathway activity scores calculated. **(C)** Expressions of TNFα in different treatments. (**P*<0.05, ***P*<0.01, ****P*<0.001, *****P*<0.0001)

**
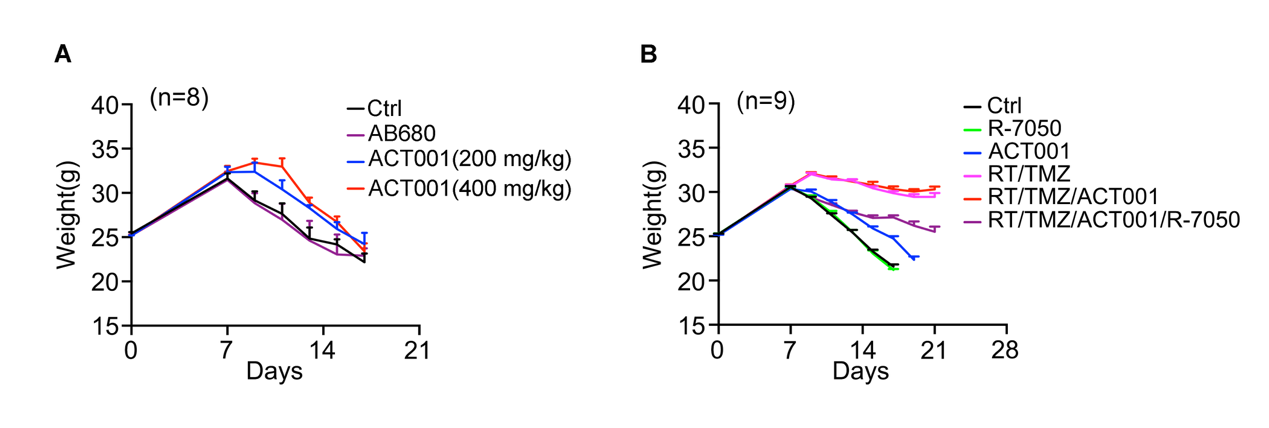
Fig. S3. Weight curves for each treatment group. (A)** Body-weight change curves of G422^TN^-GBM‑bearing mice treated with vehicle control (Ctrl), ACT001 at 200 mg/kg, ACT001 at 400 mg/kg, or AB680 (n = 8 per group). **(B)** Body-weight change curves of G422^TN^-GBM‑bearing mice treated with vehicle control (Ctrl), R-7050, ACT001 at 200 mg/kg, RT/TMZ, RT/TMZ/ACT001or RT/TMZ/ACT001/R-7050 (n = 9 per group).
